# A combination of LCPUFA ameliorates airway inflammation in asthmatic mice by promoting pro-resolving effects and reducing adverse effects of EPA

**DOI:** 10.1038/s41385-019-0245-2

**Published:** 2020-01-06

**Authors:** D. Fussbroich, R. A. Colas, O. Eickmeier, J. Trischler, S. P. Jerkic, K. Zimmermann, A. Göpel, T. Schwenger, A. Schaible, D. Henrich, P. Baer, S. Zielen, J. Dalli, C. Beermann, R. Schubert

**Affiliations:** 1 Department of Food Technology, University of Applied Sciences Fulda, Fulda, Germany; 2 Division for Allergy, Pneumology and Cystic Fibrosis, Department for Children and Adolescence, Goethe-University, Frankfurt/Main, Germany; 3 Faculty of Biological Sciences, Goethe University Frankfurt/Main, Frankfurt/Main, Germany; 4 Lipid Mediator Unit, William Harvey Research Institute, Bart’s and the London School of Medicine, Queen Mary University of London, London, UK; 5 Department of Trauma, Hand & Reconstructive Surgery, Goethe-University, Frankfurt/Main, Germany; 6 Division of Nephrology, Department of Internal Medicine III, Goethe-University, Frankfurt/Main, Germany; 7 Centre for inflammation and Therapeutic Innovation, Queen Mary University of London, London, UK

## Abstract

Lipid mediators derived from omega (n)-3 and n-6 long-chain polyunsaturated fatty acids (LCPUFA) play key roles in bronchoconstriction, airway inflammation, and resolution processes in asthma. This study compared the effects of dietary supplementation with either a combination of LCPUFAs or eicosapentaenoic acid (EPA) alone to investigate whether the combination has superior beneficial effects on the outcome of asthmatic mice. Mice were sensitized with house dust mite (HDM) extract, and subsequently supplemented with either a combination of LCPUFAs or EPA alone in a recall asthma model. After the final HDM and LCPUFA administration, airway hyperresponsiveness (AHR), bronchoalveolar lavages, and lung histochemistry were examined. Lipid mediator profiles were determined by liquid chromatography coupled with tandem mass spectrometry (LC–MS–MS). The LCPUFA combination reduced AHR, eosinophilic inflammation, and inflammatory cytokines (IL-5, IFN-γ, and IL-6) in asthmatic mice, whereas EPA enhanced inflammation. The combination of LCPUFAs was more potent in downregulating EPA-derived LTB_5_ and LTC_5_ and in supporting DHA-derived RvD1 and RvD4 (2.22-fold and 2.58-fold higher levels) than EPA alone. Ex vivo experiments showed that LTB_5_ contributes to granulocytes’ migration and M1-polarization in monocytes. Consequently, the LCPUFA combination ameliorated airway inflammation by inhibiting adverse effects of EPA and promoting pro-resolving effects supporting the lipid mediator-dependent resolution program.

## Introduction

Characterized by chronic respiratory eosinophilic inflammation, airway hyperresponsiveness (AHR), and remodeling of airways, asthma is a chronic disease affecting 300 million people worldwide.^1,2^ In recent years, lipid mediators have been shown to play an essential role in inflammatory processes in asthma.^3,4^ Pro-inflammatory lipid mediators, such as prostaglandins, leukotrienes, and thromboxanes induce bronchoconstriction and leukocyte infiltration, whereas specialized pro-resolving mediators (SPMs) downregulate infiltration, cytokine, and chemokine production and induce catabasis.^3,5,6^ Lipid mediators derive from omega (n-)3 and n-6 long-chain polyunsaturated fatty acids (LCPUFA), such as eicosapentaenoic (EPA), docosapentaenoic (n-3 DPA), docosahexaenoic (DHA), and arachidonic acid (AA). The pro-inflammatory mediators derive mainly from AA and EPA, while SPMs, such as E- and D-series resolvins, are biosynthesized from EPA, n-3 DPA, and DHA. Thus, several pro-resolving effects (e.g., the decrease of AHR and airway eosinophilia) are described for SPMs, such as RvE1, RvD1, PD1, LXA_4_ in asthma.^7–10^

As therapeutic accompaniments in asthma, n-3 LCPUFAs, such as fish oil-derivatives EPA or DHA, are suggested to be supplemented.^11^ However, there has been a lot of controversial data in recent years about the efficacy of EPA and DHA supplementations in chronic inflammation.^12,13^ One reason for the controversy may be that combinations of EPA and DHA have superior effects on the outcome of chronic inflammation compared with single-compound administrations.^14^ Consequently, we composed a LCPUFA blend and compared it with EPA supplementation alone. To prove whether the LCPUFA blend has significantly superior effects on allergic asthma, we supplemented asthmatic mice with either LCPUFA combination or EPA alone for 24 days. In contrast to EPA alone, which had adverse effects on the clinical outcome, the LCPUFA combination ameliorated the asthmatic situation significantly.

To understand why both supplementation strategies led to different results even though both contained the same amount of EPA, we determined lipid mediator profiles by liquid chromatography coupled with tandem mass spectrometry (LC–MS–MS) after supplementation was given. The results showed distinctive lipid mediator profiles for both supplementation strategies, which suggest that the combination of LCPUFA impacts lipid mediator biosynthesis and therefore, clinical outcome varies when different supplementations are given.

## Results

### A combination of LCPUFAs but not EPA alone ameliorates airway hyperresponsiveness in mice sensitized to house dust mite

**Fig. 1:**
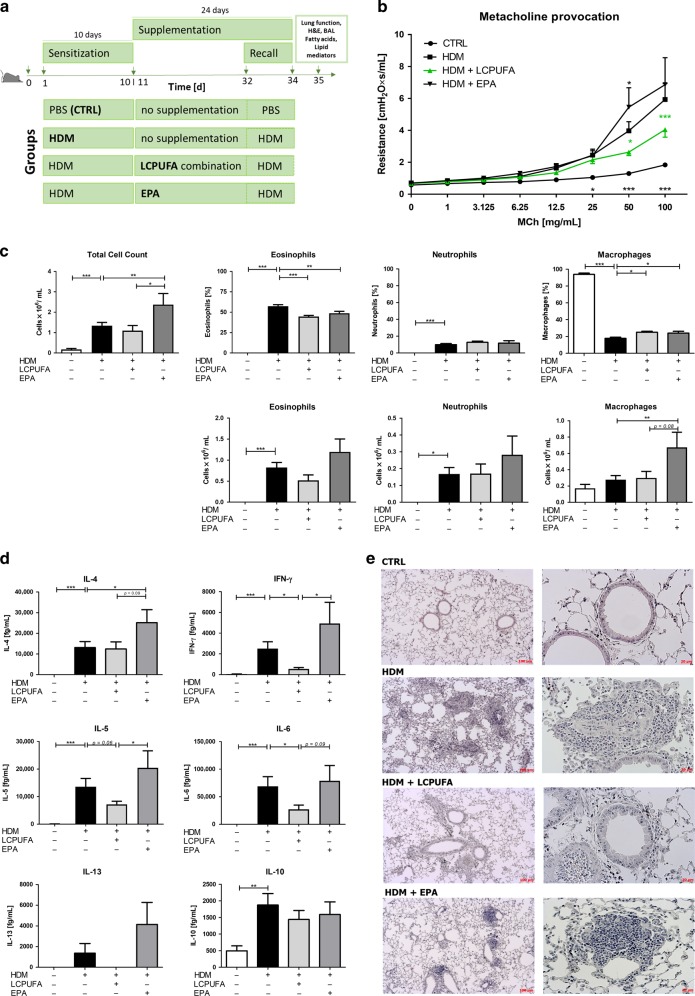
LCPUFA combination ameliorates airway inflammation in asthmatic mice. **a** Schematic design of experiments to investigate the efficacy of a LCPUFA combination versus an EPA only supplement in allergic asthma. Mice were sensitized with house dust mite (HDM) extract (or PBS as CTRL) for 10 consecutive days (d1–d10) to induce allergic asthma. Then, they either received no supplementation, a LCPUFA combination or EPA supplementation perorally for 24 days (d11–d34) on a daily basis. On the last 3 days (d32–d34), repetitive doses of HDM or PBS were administered to mice to boost the allergic sensitization (recall model ^33^). Twenty-four hours after the final HDM and LCPUFA administration, lung function measurements under MCh challenge and bronchoalveolar lavages (BALs) were conducted, and lungs were collected for H&E staining, fatty acid and lipid mediator profiling in five experimental sessions. **b** Airway resistance response following a dose-dependent methacholine (MCh) challenge (1‒100 mg/mL) in asthmatic mice (HDM) compared with control mice (CTRL) or asthmatic mice, either supplemented with a combination of LCPUFA (HDM + LCPUFA) or EPA (HDM + EPA) alone. Airway measurements were obtained using the flexiVent system (SCIREQ, Montreal, Canada). The results are expressed as mean ± SEM; *n* = 11‒15. Differences were considered statistically significant at *p*-values < 0.05. *p < 0.05, **p < 0.01, ***p < 0.001 were tested by two-way ANOVA with Bonferroni post hoc analysis (showing significances for each group compared with HDM). **c** Changes in the cytological constitution of BALs in healthy, asthmatic, and LCPUFA- or EPA-supplemented asthmatic mice. The results are expressed as mean ± SEM; *n* = 10‒20. **d** Total IL-4, IL-5, IL-13, IFN-γ, IL-6, and IL-10 cytokine levels in BALs of healthy, asthmatic, and LCPUFA- or EPA-supplemented asthmatic mice. The results are expressed as mean ± SEM; *n* = 10‒15. Differences were considered statistically significant at *p*-values < 0.05. *p < 0.05, **p < 0.01, ***p < 0.001 were tested by one-way ANOVA (each group compared with HDM) with Dunnett’s post hoc test and Student’s *t* test or the corresponding nonparametric testing. **e** H&E staining of lung tissue sections of healthy (PBS), asthmatic (HDM), or LCPUFA-supplemented asthmatic mice (HDM + LCPUFA or HDM + EPA) with magnification of ×50  and ×200. Data represent one out of *n* = 3‒5 mice per group.

After mice were sensitized with HDM or PBS as a control for 10 consecutive days, they either received no supplementation, a combination of LCPUFAs, or single EPA supplementation for 24 days (Fig. 1a). For the last 3 days of supplementation (d32–d34), mice received repetitive doses of HDM or PBS as a control. On day 35, they were then tracheotomized for lung function, and bronchoalveolar lavage (BAL) measurements followed by tissue collection.

The respiratory resistance (*R*_rs_) reflecting AHR was measured with increasing doses of methacholine (MCh) in a range of 1–100 mg/mL (Fig. 1b). AHR was reduced significantly in asthmatic mice fed with the LCPUFA combination. At 50 and 100 mg/mL MCh, AHR was significantly lower in LCPUFA-supplemented asthmatic mice compared with non-supplemented mice (50 mg/mL: HDM: 3.97 ± 0.57 cmH_2_O × s/mL; LCPUFA: 2.63 ± 0.22 cmH_2_O × s/mL (*p* < 0.05); 100 mg/mL: HDM: 5.93 ± 1.01 cmH_2_O × s/mL; LCPUFA: 4.04 ± 0.47 cmH_2_O × s/mL (*p* < 0.001)). The LCPUFA combination lowered AHR, while EPA supplementation alone did not decrease AHR in asthmatic mice. Moreover, the respiratory resistance was even increased at 50 mg/mL compared with non-supplemented asthmatic mice (HDM: 3.97 ± 0.57 cmH_2_O × s/mL; EPA: 5.45 ± 1.22 cmH_2_O × s/mL (*p* < 0.05)).

### sc-LCPUFA-supplemented asthmatic mice showed reduced airway inflammation in contrast to EPA-supplemented asthmatic mice

Immune cells were counted and differentiated in BAL fluids of control, non-supplemented asthmatic, LCPUFA- and EPA-supplemented asthmatic mice. Compared with healthy mice, sensitization with HDM led to a highly significant increase in total immune cell numbers of asthmatic mice (PBS: 0.18 ± 0.05 × 10^6^ cells/mL; HDM: 1.34 ± 0.16 × 10^6^ cells/mL, *p* < 0.001) with eosinophils from 0.00 ± 0.00% to 57.45 ± 1.84% (*p* < 0.001). Thus, confirming a significant induction of allergic asthma. Whereas LCPUFA supplementation did not change the total cell count, EPA supplementation increased the total cell counts in BALs of asthmatic mice significantly (Fig. 1c: HDM: 1.34 ± 0.16 × 10^6^ cells/mL; LCPUFA: 1.10 ± 0.25 × 10^6^ cells/mL; EPA: 2.37 ± 0.55 × 10^6^ cells/mL, *p* < 0.01). Indeed, the total cell count was 2.15-fold higher in BALs of EPA-supplemented mice compared with LCPUFA-supplemented mice. Although the relative amount of eosinophils was reduced significantly by both supplementation strategies (Fig. 1c: eosinophils: PBS: 0.00 ± 0.00%, *p* < 0.001; HDM: 57.45 ± 1.84%; LCPUFA: 44.70 ± 1.45%, *p* < 0.001; EPA: 48.80 ± 2.35%, *p* < 0.01), EPA supplementation increased the total count of macrophages in BALs of asthmatic mice significantly (Fig. 1c: macrophages: PBS: 0.17 ± 0.05 × 10^6^ cells/mL; HDM: 0.28 ± 0.05 × 10^6^ cells/mL; LCPUFA: 0.30 ± 0.08 × 10^6^ cells/mL; EPA: 0.67 ± 0.19 × 10^6^ cells/mL, *p* < 0.01).

To determine the impact of the dietary supplementation on TH1- and TH2-responses, we measured IL-4, IL-5, IL-13, and IFN-γ in the supernatants of BALs. Whereas the LCPUFA supplementation led to a significant reduction in IL-5 and IFN-γ levels compared with non-supplemented asthmatic mice, EPA-supplemented mice had significantly higher IL-4, IL-5, and IFN-γ levels compared with LCPUFA-supplemented asthmatic mice (Fig. 1d: IL-4: PBS: 0.00 ± 0.00 fg/mL, *p* < 0.001; HDM: 13132.00 ± 2892.00 fg/ml; LCPUFA: 12458.00 ± 3410.00 fg/mL; EPA: 25154.00 ± 6255.00 fg/mL, *p* < 0.05; IL-5: PBS: 59.25 ± 16.0 fg/mL, *p* < 0.001; HDM: 13574.0 ± 3020.0 fg/mL; LCPUFA: 7181.00 ± 1142.00 fg/mL, *p* *=* 0.06; EPA: 20444.00 ± 6196.00 fg/mL; IL-13: PBS: 0.00 ± 0.00 fg/mL; HDM: 1371.00 ± 934.50 fg/ml; LCPUFA: 0.00 ± 0.00 fg/mL; EPA: 4141.00 ± 2134.00 fg/mL; IFN-γ: PBS: 59.19 ± 2.29 fg/mL, *p* < 0.001; HDM: 2516.00 ± 653.10 fg/mL; LCPUFA: 548.90 ± 132.80 fg/mL, *p* < 0.05; EPA: 4934.00 ± 2043.00 fg/mL). Furthermore, we examined IL-6 which was diminished significantly by the LCPUFA combination, but almost significantly increased by EPA supplementation compared with LCPUFA-supplemented mice (Fig. 1d: IL-6: PBS: 93.25 ± 8.84 fg/mL, *p* < 0.001; HDM: 68791.00 ± 17601.00 fg/mL, LCPUFA: 27212.00 ± 7621.00 fg/mL, *p* < 0.05; EPA: 78843.00 ± 27842.00 fg/mL, *p* *=* 0.09). Of note, IL-10 levels, which were increased in asthmatic mice compared with control mice, were not significantly altered by LCPUFA or EPA supplementation (PBS: 493.40 ± 148.40 fg/mL; HDM: 1874.00 ± 345.80 fg/mL; LCPUFA: 1438.00 ± 270.5 fg/mL; EPA: 1589.00 ± 379.5 fg/mL).

Furthermore, H&E staining showed reduced eosinophilic infiltration and septa swelling in LCPUFA-supplemented asthmatic mice compared with non-supplemented asthmatic mice (Fig. 1e). In contrast to the LCPUFA combination, EPA alone promoted cell infiltration into the peribronchial regions of the lung compared with non-supplemented asthmatic mice.

### The release of EPA and n-3 DPA from cell membranes was increased by both the LCPUFA combination and EPA alone

The total and free fatty acids were determined in lungs after MCh challenge to examine (A) differences in incorporation of fatty acids after LCPUFA and EPA supplementation and (B) free fatty acid release upon MCh provocation in LCPUFA- and EPA-supplemented asthmatic mice. The total amounts of EPA and n-3 DPA were increased, and AA was reduced by both supplementation strategies (Supplementary Table 1A). Differences in the total fatty acid amount was found for DHA: The LCPUFA combination increased DHA in lungs of asthmatic mice, whereas EPA supplementation alone decreased DHA significantly. Upon MCh challenge, both supplementation strategies led to a greater release of EPA and n-3 DPA, whereas there was no significant alteration of free DHA and AA in lungs of LCPUFA- and EPA-supplemented asthmatic mice (Supplementary Table 1B).

### Lipid mediator profiling in LCPUFA and EPA-supplemented asthmatic mice

**Fig. 2:**
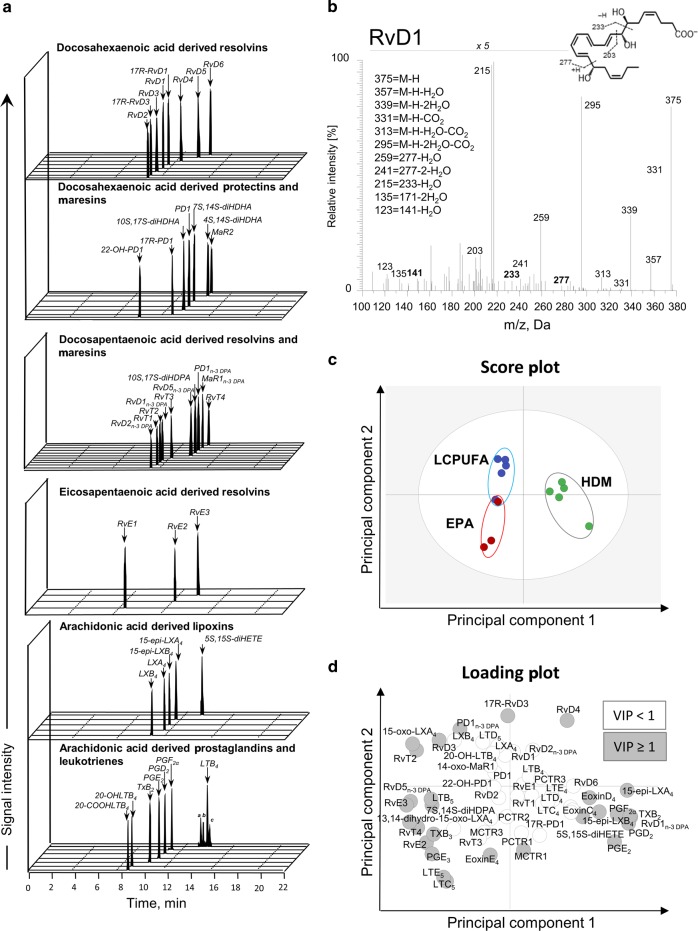
Lipid mediator identification and analysis in asthmatic mice supplemented with either a LCPUFA combination or EPA alone. Lipid mediators were identified according to (**a**) their retention times in the MRM chromatograms and their (**b**) MS/MS spectra with at least six diagnostic ions representatively shown for RvD1. Peaks in MS/MS spectra are depicted as percentage of base peak. Identification and quantification were performed by liquid chromatography coupled with tandem mass spectrometry (LC–MS–MS). Relations between the two supplementation strategies and lipid mediators were visualized by creating (**c**) score and (**d**) loading plots by partial least square projection to latent structures discriminant analysis (PLS-DA). Green = HDM; Blue = HDM + LCPUFA; Red = HDM + EPA; *n* = 3–5 mice per group. Variables with the best discriminatory power (variable importance in projection (VIP) ≥ 1) are highlighted in gray in the score plot.

Given the role of specialized pro-resolving mediators and pro-inflammatory eicosanoids, we determined the lipid mediator profiles of non-supplemented, LCPUFA- and EPA-supplemented asthmatic mice to identify the mechanisms of both supplementation strategies in order to explain the different clinical outcomes. Therefore, lipid mediators were identified and quantified by LC–MS–MS. Identification was conducted by matching retention times (Fig. 2a) to authentic and synthetic standards and to at least six diagnostic ions in the MS/MS spectra (Fig. 2b). To visualize the distinct relations of the two supplementation strategies to the lipid mediator biosynthesis, a multivariate analysis was conducted. Partial least square projection to latent structures discriminant analysis (PLS-DA) score plot (Fig. 2c) revealed that experimental groups form distinct clusters due to the different supplementation strategies. Whereas SPMs deriving from n-3 DPA and DHA were more abundant in mice fed with the LCPUFA combination, EPA and AA-derived leukotrienes were much more abundant in EPA-supplemented mice (loading plot: Fig. 2d).

### EPA- and n-3 DPA-derived SPMs were significantly elevated by both the LCPUFA combination and EPA, however, D-series resolvins (RvDs) were only maintained by the LCPUFA combination

**Fig. 3:**
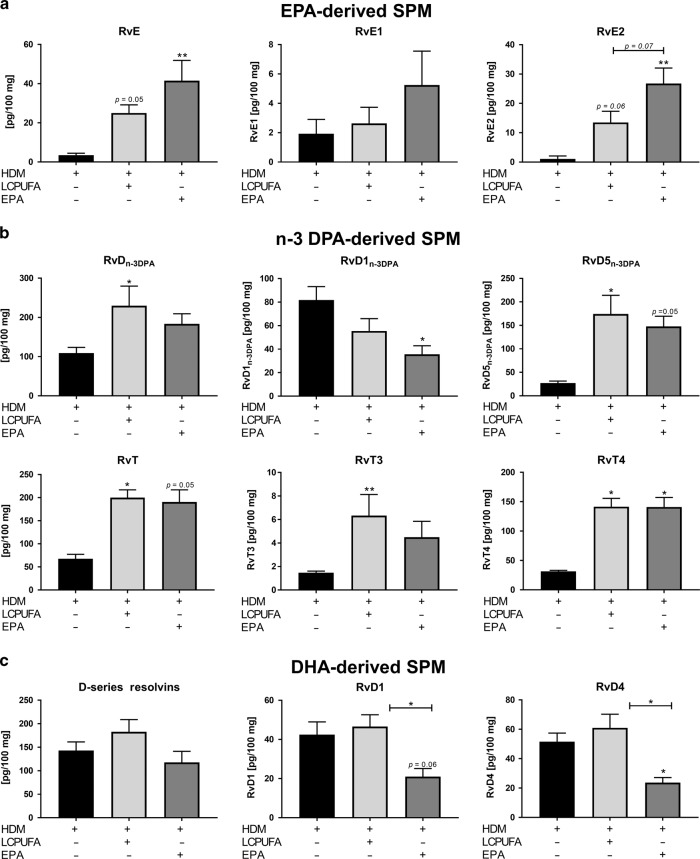
Concentrations of specialized pro-resolving mediators (SPMs) in lung tissue of asthmatic mice, which received no supplementation, the LCPUFA combination or EPA alone. Cumulative and individual levels of (**a**) EPA-derived E-series resolvins (RvE), such as RvE1 and RvE2, (**b**) n-3 DPA-derived D-series resolvins (RvD_n-3 DPA_), such as RvD1_n-3 DPA_ and RvD5_n-3 DPA_, thirteen-series resolvins (RvT), such as RvT3 and RvT4, and (**c**) DHA-derived D-series resolvins (RvD), such as RvD1 and RvD4. The results are presented as mean pg/100 mg ± SEM; *n* = 3‒5 mice per group. Differences were considered statistically significant at *p*-values < 0.05. *p < 0.05; **p < 0.01; and ***p < 0.001 tested by Kruskal–Wallis test (each group compared with HDM) with Dunn’s post hoc test and Mann–Whitney test (HDM + LCPUFA vs. HDM + EPA).

To differentiate individual changes in SPM production upon supplementation, we analyzed EPA-, n-3 DPA-, and DHA-derived SPMs (Supplementary Table 2; Fig. 3a–c). E-series resolvins (RvEs) were increased in lungs by both LCPUFA (*p* = 0.05) and EPA (*p* < 0.01) supplementation, although there were higher RvE2 levels *(p* *=* 0.07*)* in EPA-supplemented asthmatic mice than in LCPUFA-supplemented asthmatic mice (Fig. 3a). Though cumulative amounts of n-3 DPA-derived resolvins (RvDs_n-3 DPA_) (Fig. 3b) were only increased in LCPUFA- (*p* < 0.05) fed mice, which was consistent to a significantly reduced amount of RvD1_n-3 DPA_ in EPA-supplemented mice (*p* < 0.05). In contrast to RvD1_n-3 DPA_, RvD5_n-3 DPA_ was increased by both supplementation strategies (LCPUFA: *p* < 0.05; EPA: *p* = 0.05). Thirteen-series resolvins (RvTs), including RvT3 and RvT4, were elevated by both LCPUFA (*p* < 0.05) and EPA (*p* = 0.05) supplementation. While the cumulative amounts of SPMs deriving from DHA (Fig. 3c) were not altered significantly, individual RvD1 and RvD4 quantities were diminished considerably by EPA supplementation. In contrast, the concentrations of these mediators were maintained by LCPUFA supplementation. Thus, RvD1 and RvD4 levels in lungs were 2.22-fold (*p* < 0.05) and 2.58-fold higher (*p* < 0.05) in LCPUFA-supplemented asthmatic mice than in EPA-supplemented asthmatic mice.

### Both LCPUFA and EPA supplementation decreased COX-2-dependent AA-derived eicosanoids, but EPA-derived eicosanoids were biosynthesized at greater levels in EPA-supplemented mice

**Fig. 4:**
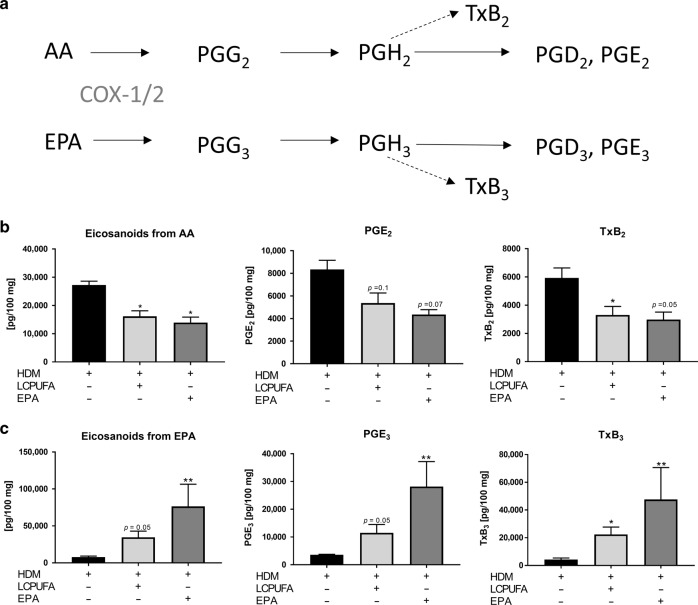
COX-2 dependant lipid mediators in the lungs of LCPUFA- and EPA-supplemented asthmatic mice. **a** Both AA and EPA are substrates for COX-2 and can be converted into prostaglandins (PG), such as PGE_2_ and PGE_3_ as well as into thromboxanes (Tx), such as TxB_2_ and TxB_3_. Individual and cumulative amounts of eicosanoids derived from (**b**) AA and (**c**) EPA in lungs of non-, LCPUFA-, or EPA-supplemented asthmatic mice. The results are expressed as mean pg/100 mg ± SEM; *n* = 3‒5 mice per group. Differences were considered as statistically significant when *p*-values < 0.05. *p < 0.05, **p < 0.01, ***p < 0.001 tested by Kruskal–Wallis test (each group compared with HDM) with Dunn’s post hoc test and Mann–Whitney test (HDM + LCPUFA vs. HDM + EPA).

Cyclooxygenase (COX)-2-dependent eicosanoids deriving from AA or EPA (Fig. 4a–c) are known to impair the inflammatory response. In our study, we observed that AA-derived mediators were reduced significantly in asthmatic mice which were supplemented with either LCPUFA (*p* < 0.05) or EPA (*p* < 0.05) compared with non-supplemented mice. These changes were mainly due to reductions in PGE_2_ and TxB_2_, which were diminished by both supplementation strategies (Fig. 4b). Furthermore, PGD_2_ was reduced in both sc-LCPUFA- (*p* = 0.1) and EPA-supplemented (*p* < 0.05) mice. In comparison, EPA-derived pro-inflammatory lipid mediators (Fig. 4c) were increased highly significant by EPA (*p* < 0.01) and almost significant by the sc-LCPUFA blend (*p* = 0.05). Thus, EPA-derived pro-inflammatory lipid mediators were 2.21-fold greater in asthmatic mice fed with EPA than in asthmatic mice being fed with LCPUFA. This was in accordance with the increased concentrations of PGE_3_ and TxB_3_, which were increased by EPA (both *p* < 0.01) more significantly than with the LCPUFA combination (*p* = 0.05; *p* < 0.05).

### The LCPUFA combination induced reduced leukotriene biosynthesis compared to EPA alone

**Fig. 5:**
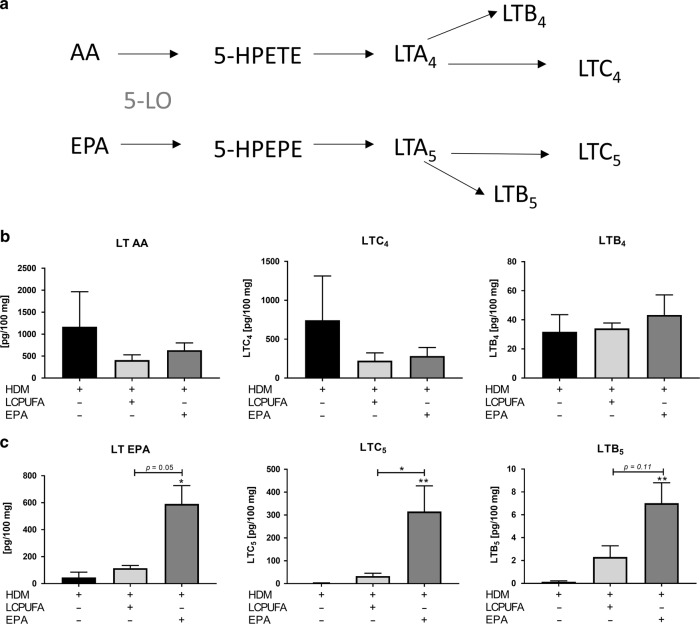
Both AA and EPA can be converted by 5-LO to monohydroperoxy 5-HPETE and 5-HPEPE which are in turn converted to leukotrienes. **a** Both AA and EPA can be converted by 5-LO over monohydroperoxy 5-HPETE and 5-HPEPE. They are biosynthesized to leukotrienes (LT) A_4_ and LTA_5_, which are then either converted into cysteinyl leukotriene LTC_4_ and LTC_5_ by glutathione-S-transferase or into LTB_4_ and LTB_5_ by hydrolases. In panels **b** and **c**, cumulative and individual amounts AA- and EPA-derived leukotrienes, such as LTC_4_, LTC_5_, LTB_4_, and LTB_5_, found in lungs of non-, LCPUFA-, or EPA-supplemented asthmatic mice are shown. The results are expressed as mean pg/100 mg ± SEM; *n* = 3‒5 mice per group. Differences were considered statistically significant at *p*-values < 0.05. *p < 0.05, **p < 0.01, ***p < 0.001 tested by Kruskal–Wallis test (each group compared with HDM) with Dunn’s post hoc test and Mann–Whitney test (HDM + LCPUFA vs. HDM + EPA).

Eicosanoids dependent on 5-lipoxygenase (LO) activity (Fig. 5a), especially cysteinyl leukotrienes, such as LTC_4_ and LTC_5,_ are particularly interesting due to their bronchoconstrictive effects in asthma. Whereas the cumulative levels of AA-derived leukotrienes (LTs) were affected equally by LCPUFA combination and EPA alone (Fig. 5b), there were significant differences for EPA-dependent leukotrienes (Fig. 5c). Cumulative levels of EPA-derived LTs were only increased by the EPA supplementation compared with non-supplemented asthmatic mice (*p* < 0.05). Thus, they were 5.13-fold higher after EPA supplementation alone compared with the LCPUFA combination (*p* *=* 0.05). This difference was in line with the significant increase of LTC_5_ (*p* < 0.01) and LTB_5_ *(p* < 0.01) in EPA-supplemented asthmatic mice compared with non-supplemented asthmatic mice. Thus, in asthmatic mice fed with EPA alone, 9.47-fold higher levels of LTC_5_ (*p* < 0.05) and 3.03-fold higher LTB_5_ levels (*p* *=* 0.11) were detected compared with the LCPUFA combination.

**Fig. 6:**
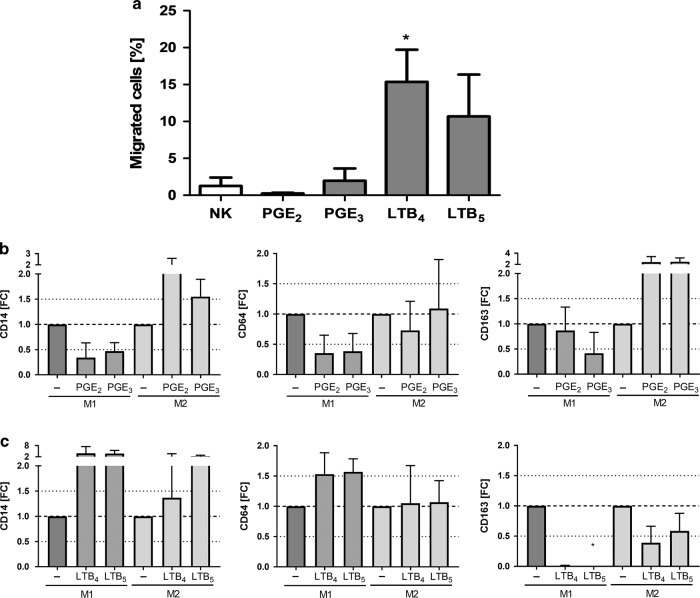
Comparison of the pro-inflammatory potency of EPA- and AA-derived prostaglandins and leukotrienes on the migration of human granulocytes and the polarization of human macrophages. **a** Chemo-attractive potency of PGE_2_ and PGE_3,_ or LTB_4_ and LTB_5_ on granulocytes. Migrated granulocytes upon either 1000 nM PGE_2,_ PGE_3,_ LTB_4_, or LTB_5_ are represented as mean (% of migrated cells relative to total number of cells) ± SEM (*n* = 6 out of *n* = 2 experiments). Impact of 1000 nM (**b**) PGE_2_, and PGE_3_ or (**c**) LTB_4_ or LTB_5_ on macrophage polarization. Cell surface marker expression of CD14, CD64 (M1), and CD163 (M2) are represented as fold changes (FC) ± SEM (*n* = 3 out of *n* = 3 experiments). Differences were considered statistically significant at *p*-values < 0.05 tested by a one-way ANOVA with Dunnett’s post hoc analysis or by a Friedman test with Dunn’s post hoc analysis.

To address the pro-inflammatory potency of EPA-derived mediators, chemotaxis assays on granulocytes and macrophage polarization assays were conducted. In Fig. 6a, the results of the chemotactic activity of PGE_2_ in comparison with PGE_3_ and LTB_4_ in comparison with LTB_5_ are depicted. PGE_2_ as well as PGE_3_ did not promote granulocytes recruitment. In contrast, LTB_4_ (*p* < 0.05*)* significantly increased granulocyte recruitment at 1000 nM, whereas LTB_5_ increased migration only tendentiously. However, no statistically significant differences in recruitment of granulocytes between PGE_2_ and PGE_3_ as well as LTB_4_ and LTB_5_ were observed. We next tested the ability of PGE_2_ and PGE_3_ on macrophage polarization. Regarding the impact of PGE_2_ and PGE_3_ on macrophage polarization, both of these mediators reduced CD14 expression in macrophages schewed toward an M1 phenotype using GM-CSF, IFN-γ, and LPS. On the other hand, PGE_2_ and PGE_3_ promoted CD14 in macrophages differentiated toward an M2 phenotype using M-CSF and IL-4. These mediators also reduced the expression of CD64 (M1-marker) in M1-differentiated macrophages and upregulated CD163 (M2-marker) in M2-schewed macrophages. These findings suggest that both lipid mediators display anti-inflammatory actions in regulating macrophage polarization (Fig. 6b; Supplementary. Fig. 3C). In contrast, LTB_4_ and LTB_5_, equally increased CD14 in M1- and M2-induced macrophages, increased M1-markers, such as CD64, in M1-induced macrophages and reduced M2-markers, such as CD163 (LTB_5_: *p* < 0.05), in M2-induced macrophages (Fig. 6c; Supplementary. Fig. 3).

**Fig. 7:**
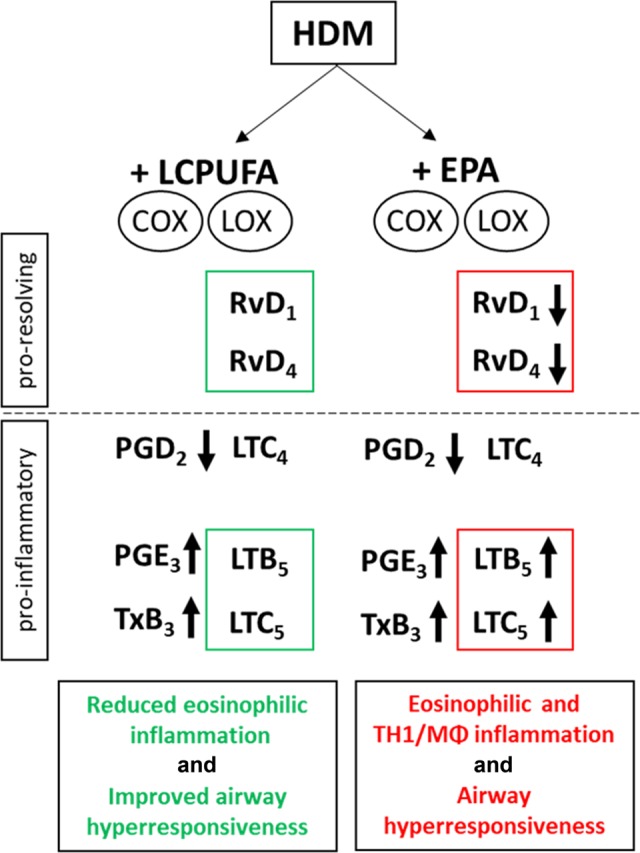
Main differences in lipid mediator profiles of HDM-induced asthmatic mice supplemented with either the LCPUFA combination or EPA alone, suggesting mechanisms for different clinical outcomes. Whereas RvD1 and RvD4 were biosynthesized at significantly lower levels in EPA-supplemented mice, pro-inflammatory eicosanoids, such as LTB_5_ and LTC_5_, were significantly higher in EPA-supplemented mice.

In summary, EPA supplementation led to significant lower RvD1 and RvD4 levels, whereas the LCPUFA combination maintained these SPMs (Fig. 7). Furthermore, EPA-derived 5-LO products, such as LTB_5_ and LTC_5_ were increased in EPA supplemented but not LCPUFA-supplemented asthmatic mice underlining the adverse effects of single EPA supplementation.

## Discussion

Dietary supplementations with long-chain polyunsaturated fatty acids, predominantly fish oil-associated eicosapentaenoic (C20:5n-3) and docosahexaenoic acids (C22:6n-3), are being suggested as a supportive treatment strategy in chronic inflammatory diseases, such as asthma.^11,14,15^ Given the role of LCPUFA-derived pro-inflammatory eicosanoids and SPMs on AHR, leukocyte infiltration, and resolution of inflammation,^3,5,16^ LCPUFA supplementation has a significant impact on the clinical outcome of asthma. To investigate whether a LCPUFA combination is superior to EPA supplementation alone, lung function measurements, histological, and BAL analyses were conducted in an allergic asthma mouse model. Furthermore, pro-inflammatory eicosanoids and pro-resolving mediators in the lungs of these mice were analyzed, to determine mechanisms for lipid mediator biosynthesis in response to each supplementation strategy.

The LCPUFA combination ameliorated AHR during MCh challenge and improved eosinophilic cell infiltration in lung tissue. In contrast, EPA increased cellular and cytokine-driven inflammation and impaired lung function. The cellular increase was mainly due to macrophages, which were concordant with elevated IFN-γ and IL-6 levels. Macrophages play an important role in maintaining and resolving inflammation, especially in tissues.^16,17^ Following the dichotomic subtype classification of macrophages in asthma into pro-inflammatory M1 and anti-inflammatory M2 macrophages,^17^ our results suggest the macrophages to be pro-inflammatory M1 macrophages, as deduced from elevated IL-6 and IFN-γ and unaltered IL-10 levels. Regarding the reduction of IL-5 and IL-13, it is also discussable that lung type 2 innate lymphoid cells (ILC2s) might have been involved. ILC2s were nicely shown to be activated by leukotrienes, such as LTC_4_ and LTB_4_ and to be inhibited by PGE_2_.^18,19^ Thus, it is discussable that they might have contributed to the worsening of the clinical outcome in EPA-supplemented asthmatic mice. However, we focused on the increased macrophage infiltration upon EPA supplementation, because this was one obvious finding associated with the deterioration of asthmatic mice under EPA supplementation.

To get deeper insights into the mechanisms of lipid mediator programs and to understand why the supplementation strategies led to different clinical outcomes, we determined lipid mediator profiles by LC–MS–MS. We found that both supplementation strategies increased EPA- and n-3 DPA-dependent SPM levels, which were in line with the free fatty acid levels. Haworth et al. administered 100 ng/d RvE1 to asthmatic mice for three consecutive days, and showed a significant reduction in AHR at 100 mg/ mL MCh and a distinctive reduction of eosinophil and macrophage infiltration.^7^ Although we measured significant increases in E-series resolvins in EPA-supplemented asthmatic mice, we could not observe an amelioration of AHR and eosinophilic infiltration. One reason for this might be the fact that we did not apply RvE1 itself, and that EPA supplementation led not only to increased RvE levels but also to diminished levels of total DHA, which is a precursor for SPMs, such as RvD1 and RvD4. Compared with LCPUFA-supplemented asthmatic mice, RvD1 and RvD4 levels were 2.22-fold and 2.58-fold lower in the EPA group. In contrast to EPA alone, the LCPUFA combination maintained RvD1 and RvD4 levels, which was probably due to the adding of DHA to the LCPUFA blend. Titos et al. found that DHA and RvD1 induce a phenotypic switch in macrophages from a pro-inflammatory classical activation profile toward an alternative anti-inflammatory M2-like state.^20^ Therefore, EPA alone might have been less effective at inducing the macrophage phenotypic switch compared with the LCPUFA supplementation. This is consistent with Li et al.^21^ who demonstrated that n-3 LCPUFA intake is inversely longitudinally associated with the incidence of asthma, but DHA administration has a greater inverse association than EPA. In addition, Rogerio et al.^8^ showed reduced lung resistance under methacholine challenge with 10 ng of RvD1, which is one-tenth of the amount of RvE1 Haworth et al.^7^ administered. These results emphasize the efficacy and significant roles of DHA-derived D-series resolvins in asthma. Notably, RvD1 has been shown to counter-regulate COX-2 and the translocation of 5-LO, which lead to diminishing pro-inflammatory mediators in chronic inflammation.^22,23^

In view of COX-2- and 5-LO-derived pro-inflammatory lipid mediators, EPA supplementation might have caused the deterioration of the asthmatic situation due to EPA being an alternate substrate for both enzymes. Whereas both LCPUFA and EPA supplementations decreased AA-derived PGE_2_, TxB_2_, as well as PGD_2_ on one hand, we observed that EPA supplementation led to a significant increase in PGE_3_ and TxB_3_ on the other hand. In ex vivo experiments, PGE_3_ had no significant impact on the infiltration of granulocytes, whereas LTB_5_ did tendentiously increase granulocyte migration (Fig. 6a). However, PGE_3_ similarly displayed anti-inflammatory potencies to PGE_2_ in regulating macrophage polarization (Fig. 6b). Thus, an increase of PGE_3_ is not likely to explain a further infiltration of cells into the lungs of EPA-supplemented mice. Furthermore, significantly higher proportions of EPA were converted to pro-inflammatory mediators in EPA-supplemented asthmatic mice, suggesting that the LCPUFA blend was more efficient in counter-regulating COX-2 than EPA alone.

Similar to COX-2 products, the 5-LO products (LTC_5_ and LTB_5_) were found at significantly higher levels in EPA-supplemented mice than in non- and LCPUFA-supplemented asthmatic mice. Particularly, LTC_5_ was increased significantly higher (9.47-fold higher, *p* < 0.05) in EPA-supplemented mice than in the LCPUFA group. In contrast to LTB_5_, which is according to literature only ~5–10% as active as LTB_4_,^24^ the 5-series cysteinyl leukotrienes LTC_5_, LTD_5_, and LTE_5_ are comparable with their AA-derived congeners, such as LTC_4_, in evoking nonvascular smooth muscle contraction.^24–26^ This would explain why we did not observe any amelioration of AHR in asthmatic mice fed with EPA. Thien et al.,^27^ who incubated human eosinophils with either EPA or AA, observed that total LTC generation was nearly twofold higher in cells incubated with EPA than in cells incubated with AA. Thus, their results suggest that EPA does not inhibit the biosynthesis of cysteinyl leukotrienes but actually stimulates their biosynthesis at lower doses than AA.^27^ Furthermore, Chapkin et al.^28^ found that n-3 LCPUFAs are incorporated differentially into macrophage phospholipid subclasses after dietary fish oil supplementation. In addition, they found that macrophage peptidoleukotriene synthesis was also strongly influenced by the fish oil supplementation resulting in a significantly higher LTC_5_/LTC_4_ ratio. With regards to increased LTB_5_-levels in EPA-supplemented mice, our in vitro experiments demonstrated that LTB_4_ significantly increased the recruitment of granulocytes, and LTB_5_ tendentiously increased migration of granulocytes (Fig. 6a). However, LTB_5_ was as potent as LTB_4_ in promoting M1-polarization and diminishing M2-polarization in monocytes (Fig. 6c). Thus, the increased levels of LTB_5_ might have led to further M1-macrophage infiltration into the lungs of EPA-supplemented asthmatic mice. Therefore, increased LTC_5_- and LTB_5_-levels are likely to play a major role due to which we observed a promotion of airway inflammation in asthmatic mice fed with EPA, instead of any amelioration of AHR.

It is feasible that significantly lower levels of RvD1 and RvD4 in EPA-supplemented mice have led to higher 5-LO translocations, and subsequently these have led to an increased cysteinyl leukotriene biosynthesis. In contrast, LCPUFA combinations might feature significantly different effects than individual LCPUFAs, such as EPA, due to competitive substrate inhibition of enzymes, altered carnitine transport, and modifications of distinct enzymes involved in fatty acid and lipid mediator biosynthesis.^15,23,28–31^ Thus, LCPUFA combinations might exhibit beneficial synergistic effects, which positively influence clinical outcomes.^15^ Supplementation of a n-3 and n-6 LCPUFA combination in humans has been shown to reduce bronchial inflammation after low-dose allergen challenge.^32^ Schubert et al.^32^ found that serum eosinophils, eosinophilic cationic protein, and in vitro cysteinyl leukotrienes were significantly reduced in asthmatic patients supplemented with a combined LCPUFA blend. Thus, our data support the hypothesis of synergistic n-3 and n-6 LCPUFA combination effects,^15^ concerning the restoration of fatty acid homeostasis in cellular membranes, modifying lipid mediator biosynthesis pathways, and controlling inflammatory processes by focusing on resolution of inflammation in the bronchoalveolar system.

In summary, LCPUFA supplementation led to an amelioration of AHR as well as a reduction of eosinophilic cell infiltration and remodeling of airways. In contrast, EPA supplementation caused further infiltration of pro-inflammatory cells (i.e., macrophages) into the lung. The LCPUFA combination was more potent in downregulating EPA-derived pro-inflammatory mediators, such as LTB_5_ and LTC_5_, and in supporting SPM biosynthesis. Consequently, we demonstrated that a LCPUFA combination is more effective in limiting and resolving inflammation in allergic asthma than a single EPA administration by promoting pro-resolving effects and reducing EPA-derived pro-inflammatory effects. Thus, we recommend the supplementation of LCPUFA combinations to support asthma control.

## Methods

### Animal care

Female C57BL/6 mice were purchased from Charles River Laboratories (Wilmington, Delaware) at the age of 6–8 weeks and maintained in stainless steel cages under sterile conditions with an alternating 12 h light/dark cycle at the university hospital in Frankfurt. Mice were fed with water and lab chow (Ssniff, Soest, Germany) with 6% fat content (C 14:0: 0.01%; C 16:0: 0.68%; C 16:1: 0.04%; C 18:0: 0.22%; C 18:1: 1.44%; C 18:2: 3.21%; C 18:3: 0.37%; C 20:0: 0.03%; C 20:1: 0.01%) *ad libitum*. All animal procedures were performed according to protocols approved by the German Animal Subjects Committee (Gen.Nr.FK/1036).

### Allergic asthma mouse model

Allergic asthma was induced by sensitization with 40 µg house dust mite extract (HDM; *Dermatophagoides pteronyssinus*, Greer, New York City, New York) or with PBS (phosphate-buffered saline, pH = 7.4, Life Technologies, Darmstadt, Germany) as control given on a daily basis for 10 consecutive days (Fig. 1a). To suppress the swallowing reflex, mice were anesthetized with isoflurane (initial dose: 5%, maintenance dose: 2.5%, Baxter, Unterschleißheim, Germany). Subsequently, mice have received either 25 µL of PBS or 25 µL of HDM (1.6 mg/mL in PBS) intranasally. After 21 days of rest, mice were given repetitive doses of 40 µg HDM (or PBS as control) for another 3 consecutive days (as described in the recall model^33^). All mouse experiments were performed in five experimental sessions, in which we measured lung function, collected BAL fluids and lungs for further measurements, such as H&E staining and lipid mediator measurements.

### Dietary supplementation

Dietary supplements were applied orally to each mouse for 24 days starting on day 11 (Fig. 1a). EPA supplementation contained 1000 mg/kg/day EPA. The LCPUFA combination contained 1000 mg/kg EPA, 229.6 mg/kg DHA, 246.0 mg/kg GLA, and 200.9 mg/kg SDA. The LCPUFA combination or EPA were mixed freshly on a daily basis and diluted in 0.5% (w:v) gum arabic solution (gum arabic powder, Carl Roth, Karlsruhe, Germany; sterile water) to a final volume of 200 µL per dose, which was inevitable to assure emulsification and a defined application volume. Emulsions were homogenized using an ultrasonic homogenizer (Sonopuls, Bandelin, Berlin, Germany) and they were applied immediately to the mice via a feeding needle (canula, 0.70 × 30 mm, LL, curved, Knopf C, Robert Helwig GmbH, Berlin, Germany), which was connected to a 1 -mL syringe (Becton Dickinson, Frankfurt/Main, Germany).

### Lung function

Twenty-four hours after the final administration of HDM to the asthma mice or PBS to the control mice, all mice were anesthetized with Xylazine (10 mg/kg; Bayer Vital, Leverkusen, Germany) and Ketanest^®^ (100 mg/kg; CuraMed GmbH, Karlsruhe, Germany). Thereafter, they received another anesthesia with lidocaine (Xylocaine Pumpspray, 10 mg/spray burst, Astra Zeneca, Wedel, Germany) and became tracheotomized. The trachea was cannulated by insertion of a tubing adapter (18 G, BD, Franklin Lakes, New Jersey), which was fixed by a suture. Immediately after this, mice were attached to the tubing of the lung function measurement device for ventilation. Lung function was measured with a flexiVent FX system with an integrated FX1 module (Scireq, Montreal, QC, Canada). For methacholine challenge, a small particle aeroneb nebulizer (aeroneb ultrasonic nebulizer, Scireq, Montreal, QC, Canada) was attached to the FX1 module leading to Y-tubing for ventilation (volume: 10 mL/kg and frequency: 2.5 Hz). The ventilation reached 150 breaths/min. After a short ventilation period (Mouse Default CMV), the script for dose–response studies (Mouse IV Dose Response rel. B) was started, and deep inflation (v7.0) was performed to enlarge the lungs up to a pressure of 27 cmH_2_O in order to build stable experiment conditions by restoring airway patency and normalizing lung volume. Thereafter, single frequency forced oscillation (SnapShot-150 v7.0) and broadband forced oscillation (Quick Prime-3 v7.0) were performed automatically by the software. Three consecutive SnapShot and Quick Prime perturbations and one deep inflation perturbation were undertaken prior to methacholine (MCh) challenge. Subsequently, 12 measurements of SnapShot and Quick Prime perturbations for each MCh concentration were conducted with increasing concentrations of MCh (1; 3.125; 6.25; 12.5; 25; 50; and 100 mg/mL; w:v in PBS). Changes in respiratory resistance (*R*_rs_) were calculated from peak values of Snapshot measures after each dose.

### Bronchoalveolar lavage (BAL)

Following lung function measurements, bronchoalveolar lavages were conducted by insertion of a cannula (Braunüle, 21 G, shortened to 2 cm, B. Braun, Melsungen, Germany) into the trachea which was fixed by a suture. BAL fluids were taken by slow injection and subsequent aspiration of 1 mL PBS-EDTA solution (0.5 mM EDTA; (w:v); Merck, Darmstadt, Germany) with a 1 -mL syringe. Three aliquots were collected and stored on ice until further preparation.

Subsequently, aliquots were centrifuged, and the supernatants were collected for cytometric bead array (CBA) measurement. Cell pellets were pooled and incubated with ACK lysis buffer (Sigma-Aldrich, Taufkirchen, München, Germany) for 3 min to lyse any remaining erythrocytes. The lysis reaction was stopped with PBS, and supernatants were discarded after centrifugation. Subsequently, pellets were diluted in PBS, pooled, and cells were counted with the Trypanblue method. Sixty thousand cells were placed in cytospin holders (Single Cell Funnels, Tharmac, Waldsolms, Germany) and applied to microscope slides using cytocentrifugation (450 rpm, 6 min.; Cytospin 3, Shandon, Thermo Fischer Scientic, Dreieich, Germany). After at least 12 h of drying, slides were stained using Pappenheim’s stain (Giemsa’s azur eosin and May-Grünwald’s modified eosine methylene blue solution, Merck, Darmstadt, Germany) and covered with Eukitt^®^ (Sigma-Aldrich, Taufkirchen, Germany). Differentiation of cells was conducted by counting 400 cells per slide using a light microscope (Leica, DME, Wetzlar, Germany).

### H&E staining

After flushing with PBS to remove erythrocytes, lungs were perfused via the trachea with 4% paraformaldehyde (w:v; in PBS) for a minimum of 10 min and then stored for 24 h in 4% paraformaldehyde. Thereafter, lungs were transferred to embedding cassettes (Histosette 1, Simport, Bernard-Pilon, Canada), washed in water, and stored in 100% ethanol (Sigma-Aldrich, Taufkirchen, Germany) until they were completely dehydrated. Dehydrated lungs were embedded in paraffin using embedding forms (Medite online, Burgdorf, Germany) and an embedding station (Medax, Salt Lake City, Utah). After storing paraffin-embedded lungs for at least 2 h at −30 °C, 10 -µm lung tissue sections were sliced using a Cryotom (CM3050S, Leica, Wetzlar, Germany). Lung sections were placed on a microscope slide (Superfrost^®^, Sondheim v. d. Rhön, Germany), smoothed in a water bath at 40 °C, and then dried for at least 30 min at room temperature (RT). Staining was performed using hematoxylin for 10 min (ready to use, Applichem, Darmstadt, Germany) and eosin G for 3 min (Carl Roth, Karlsruhe, Germany; acidified with glacial acetic acid). Subsequently, slides were treated with increasing ethanol concentrations and xylol (Fischer Scientific, Dreieich, Germany) and then covered with Histofluid (Marienfeld, Lauda-Königshofen, Germany). Eosinophil and macrophage infiltration were evaluated using an Observer Z1 microscope with AxioCamMRm, (Zeiss, Jena, Germany) and AxioVisionRel 4.72 software.

### Cytokine measurement

The concentrations of IL-5, IFN-γ, IL-6, and IL-10 were examined in one aliquot of BAL supernatants using the BD™ CBA Mouse Enhanced Sensitivity Flex Set System and BD™ CBA Mouse Enhanced Sensitivity Master Buffer Kit (both BD Bioscience, Franklin Lakes, New Jersey). Each BD™ CBA Flex Set contained one bead population with a distinctive fluorescence intensity, as well as the appropriate phycoerythrin (PE) detection reagent and standard. Frozen supernatants were thawed at RT and centrifuged at 10,000 rpm for 10 min at 2 °C. In total, 50 µL of Flex Set Standards (0.274–200 pg/mL) or samples were incubated with 20 µL capture bead mixture in 96-well plates (filter plates, Millipore MultiScreen_HTS_-BV 1.2 µm, Merck, Darmstadt, Germany) in the dark at RT for 2 h. Then, 20 µL of detection reagent A was added and incubated for 2 h. After incubation, filter plates were washed using a vacuum manifold (Millipore MultiScreen_HTS_ Vacuum Manifold, Merck, Darmstadt, Germany), and 20 µL of detection reagent B were added and incubated for 1 h. Following three washes and the addition of 150 µL wash buffer, tests were performed using a BD FACS Verse™ (BD Biosciences, Franklin Lakes, New Jersey) and analyzed by FCAP Array v3 Software (BD Biosciences, Franklin Lakes, New Jersey). IL-4 and IL-13 were examined in another aliquot of the same supernatants of BAL fluids used for IL-5, IFN-γ, IL-6, and IL-10 using LEGENDplex^TM^ Mouse Kit including appropriate beads, detection antibodies, and standards (BioLEGEND, San Diego, USA). In all, 25 µL of each standard or sample was mixed 1:2 (v:v) with wash buffer in a 96-well plate, and then transferred into a 96-well filter plate. Afterwards, 25 µL of capture beads were added to each well. The plate was then sealed and placed onto a shaker for 2 h. After incubation, each well of the filter plate was washed with 200 µL wash buffer two times using a vacuum manifold (Millipore MultiScreen_HTS_ Vacuum Manifold, Merck, Darmstadt, Germany). Then, 25 µL of detection antibodies were added to each well, and plate was sealed and placed on a plate shaker for another hour followed by the addition of 25 µL SA-PE and another 30 min of shaking. After washing two times, 150 µL wash buffer were added to resuspend the beads on the plate shaker for 1 min. Measurements were performed using BD FACS Verse^TM^, as described above.

### Lipid mediator profiling

Lungs were collected immediately from killed mice, snapped frozen in liquid nitrogen, and stored at −80 °C until further sample processing. Tissue was quenched in ice-cold methanol containing 500 pg of each deuterated (d) internal standard (d_8_-5S-hydroxyeicosatetraenoic acid, d_4_-LTB_4_, d_5_-LXA_4_, d_4_PGE_2_, and d_5_-RvD2) using a plastic rod. Samples were centrifuged, and one aliquot of the supernatant was used for solid-phase extraction using ExtraHera (Biotage), as previously described.^34^ Identification and quantification were performed using LC–MS–MS, (Sciex 5500 and 6500+, Warrington, UK) acquiring parent and characteristic daughter ions via multiple reaction monitoring after either negative or positive electrospray ionization. Lipid mediators were identified according to their retention time and by at least six diagnostic ions in the mass spectra as further described by Dalli and Serhan.^34^ Partial least square projection to latent structures discriminant analysis (PLS-DA) was performed using SIMCA 14.1 software (Umetrics, Umea, Sweden). Evaluation of loading plots was performed as previously described,^35^ and variables with best discriminatory power (variable importance in projection ≥ 1) were highlighted in the Score plot.

### Chemotaxis assay

Human granulocytes were obtained by ficoll separation and erythrocyte lysis with 1× red blood cell lysis solution (Miltenyi Biotec, Bergisch Gladbach, Germany). After washing twice with PBS, cell viability and cell count were assessed by standard 0.5% (w:v) trypan blue cell staining (Biochrom, Berlin, Germany). For chemotaxis experiments, the wells of ChemoTx^®^ Plates (NeuroProbe, Gaithersburg, USA; 96-well plates, with 5 -µm pore size, 5.7 -mm filter in diameter, 300 -µL capacity) were filled with 300 µL of chemotactic reagents dissolved in migration buffer (Hanks' solution with 20 mM HEPES and 0.1% BSA at pH = 7.6 (all purchased from Sigma-Aldrich, Taufkirchen, Germany)). Chemotactic reagents PGE_2_, PGE_3_, LTB_4_, and LTB_5_ were all purchased from Cayman Chemicals (Ann Arbor, USA), which were initially dissolved in ethanol and then diluted with migration buffer to 1000 nM. Eosinophilic cells were diluted to 6 × 10^6^ cells/mL in migration buffer, and 50 µL of the cell suspension (300.000 cells) were placed on top of the filters. After 2 h at 37 °C incubation time, cells on the top of the filter were discarded, and cells in the solutions of the lower chambers were counted for 60 s with a rapid flow rate using the FACSVerse flow cytometer (BD Biosciences, Heidelberg, Germany) and the FACSuite software v1.0.6 (BD Biosciences, Heidelberg, Germany).

### Statistics

Data are displayed as mean ± standard error of the mean (SEM), and were evaluated using GraphPad Prism 5 and 7 (GraphPad Software, La Jolla, CA). Comparisons between more than two groups and repeated measures (MCh challenge, Fig. 1b) were analyzed using a two-way ANOVA with Bonferroni post hoc analysis. Comparisons between more than two groups without repeated measures were performed by using one-way ANOVA with Dunnett’s post hoc analysis against the disease group (HDM). Comparisons between more than two groups with repeated measures were performed by using Friedman’s test with Dunn’s post hoc analysis against the control group. In addition, unpaired *t* tests were conducted to find differences between HDM + LCPUFA and HDM + EPA. In case of not normally distributed data or samples sizes less than five, we used the corresponding nonparametric testing. Differences were considered statistically significant at *p* < 0.05.

## Supplementary information

Supplementary Information
