# Supplementary material for: A combination of LCPUFA ameliorates airway inflammation in asthmatic mice by promoting pro-resolving effects and reducing adverse effects of EPA
Source: Mucosal Immunol. 2020 Jan 6;13(3):481–92. doi: 10.1038/s41385-019-0245-2 (PMC7181394; doi:10.1038/s41385-019-0245-2)
Supplement: Supplementary file 1 — Supplementary Information [file 41385_2019_245_MOESM1_ESM.pdf]

Supplementary Figure 1

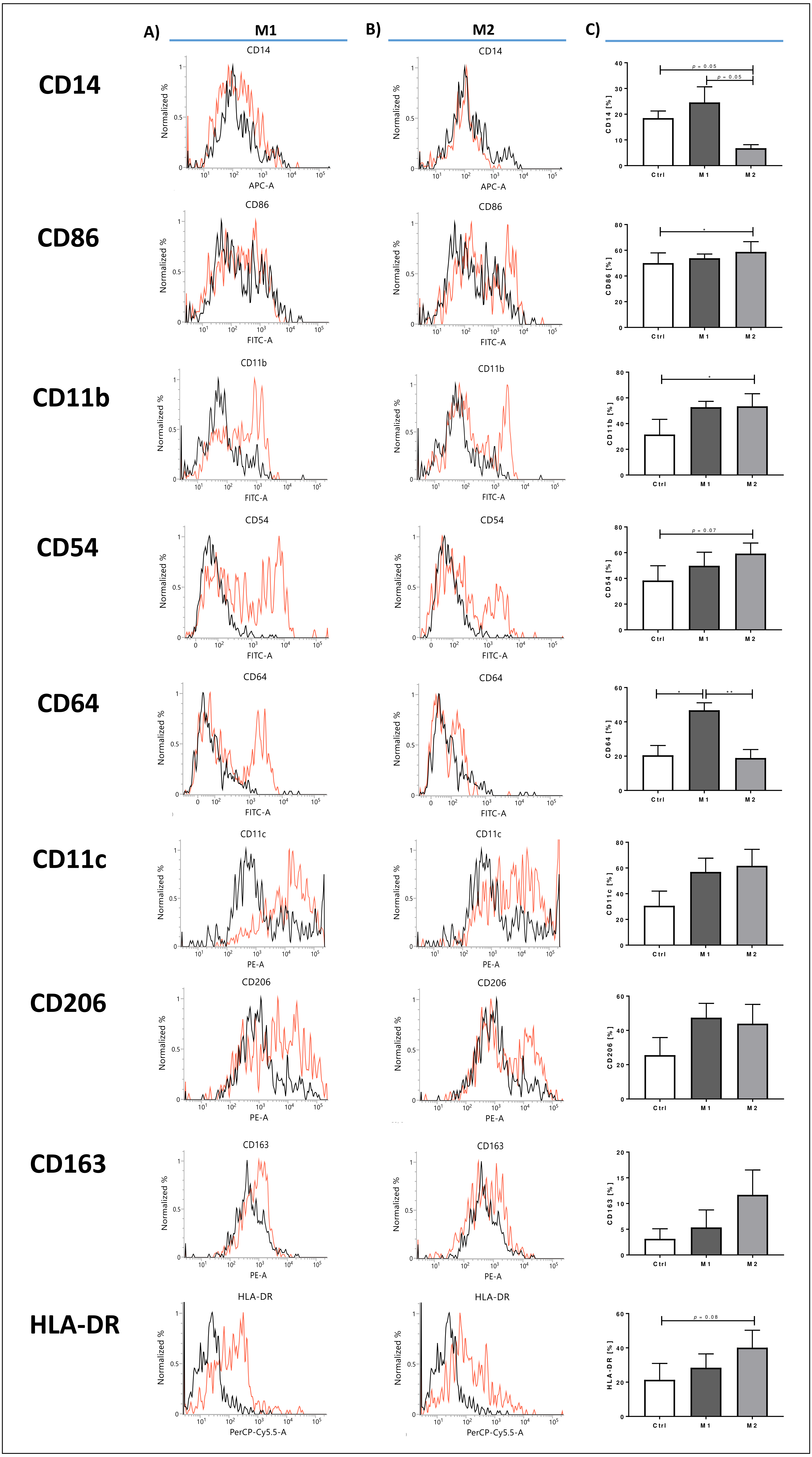

Supplementary Figure 2

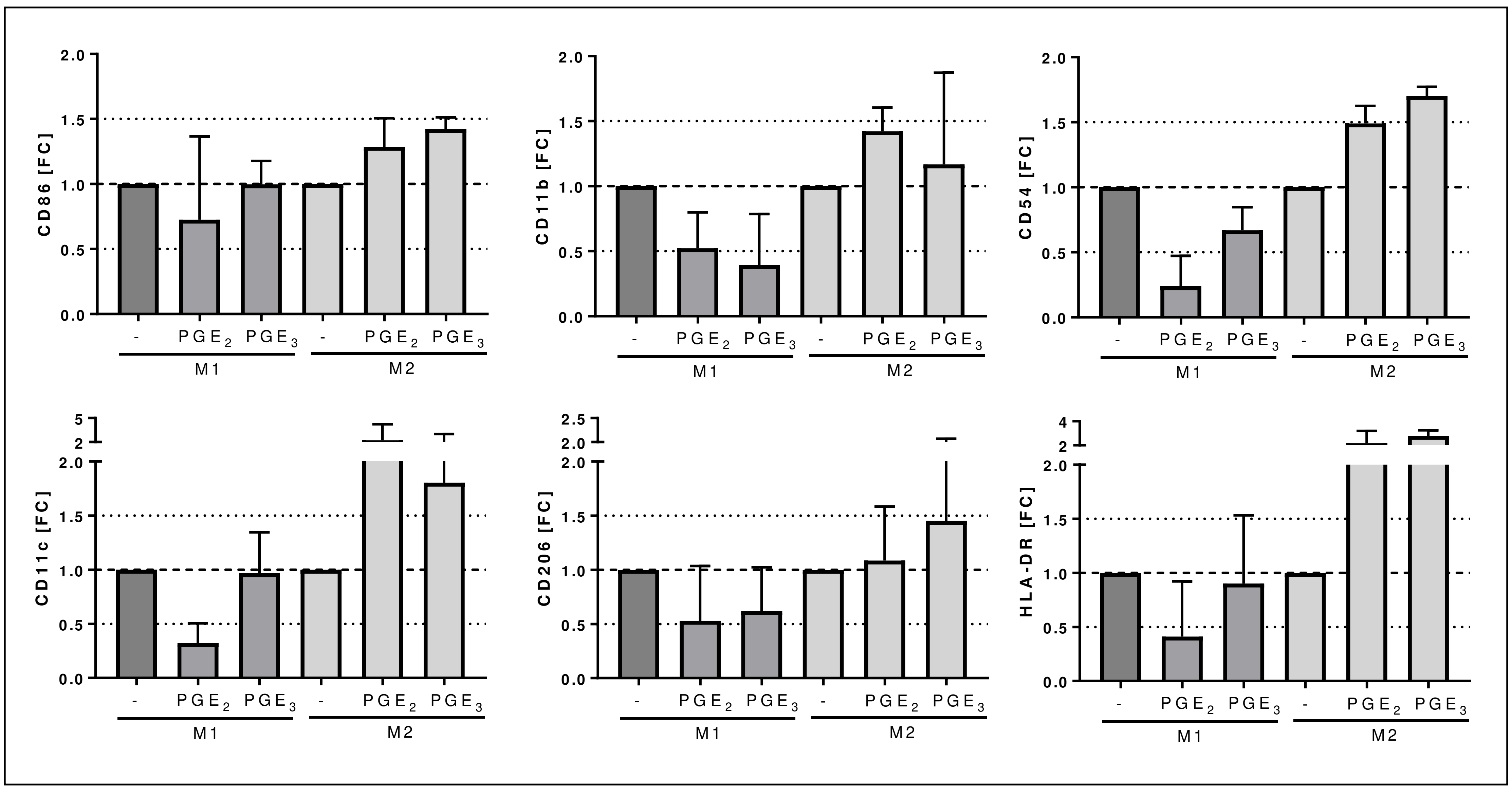

Supplementary Figure 3

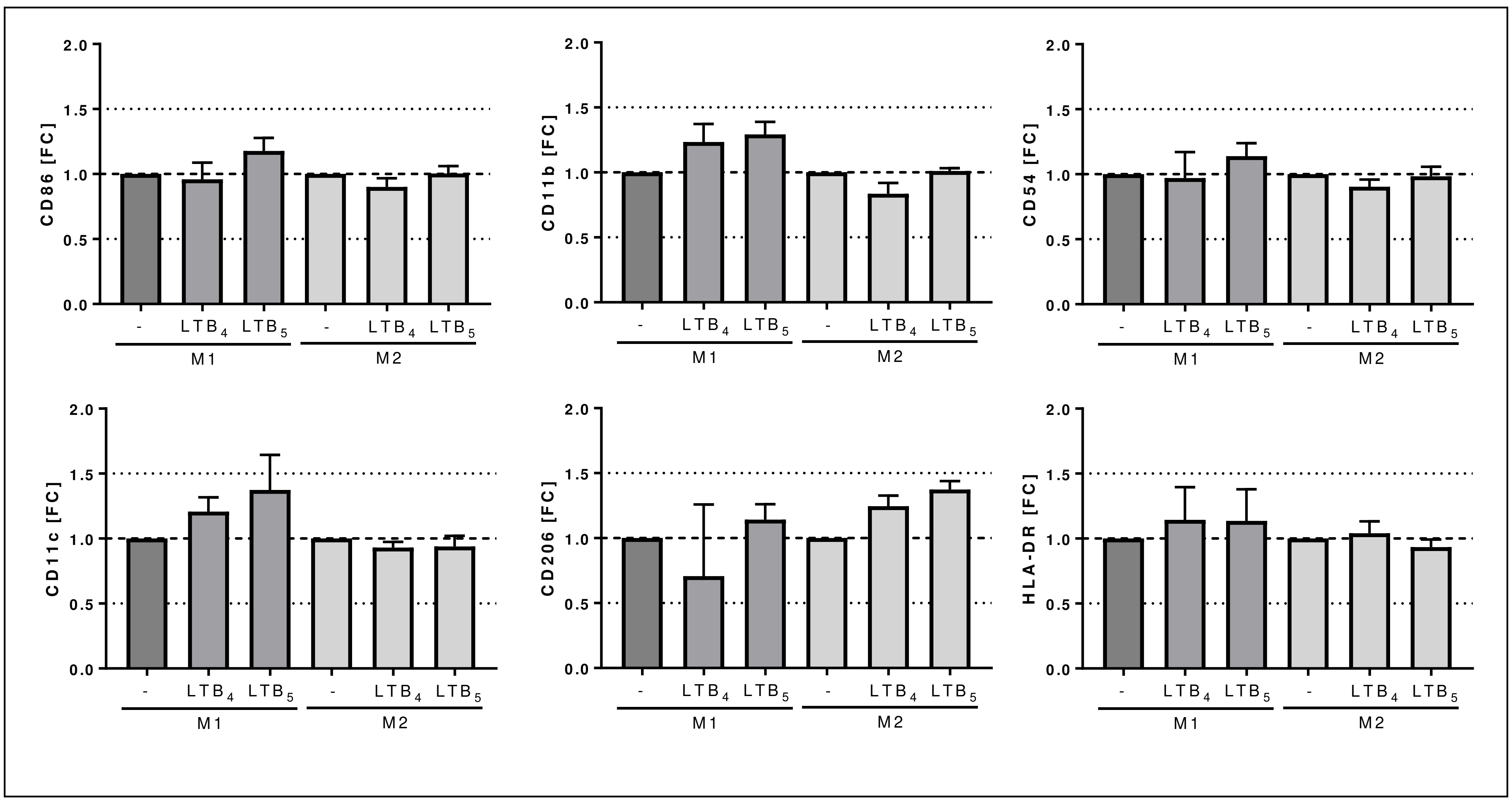

Supplementary

Methods: Total fatty acid examination

Lung tissue was homogenized and erythrocytes were lysed with ACK lysis buffer (Ammonium-Chloride-Potassium, Sigma-Aldrich, Taufkirchen, Germany). Subsequently, fatty acids were extracted according to Bligh and Dyer (32) and derivatized according to Kohn et al. (33). After extraction with chloroform, methanol (both Sigma-Aldrich, Taufkirchen, Germany) and water, the lipid fraction was completely dried by evaporation (Liebisch Labortechnik GmbH, Bielefeld, German) under nitrogen (AlphaGaz 1, Air Liquide Deutschland GmbH, Düsseldorf, Germany). For derivatization, extracted fatty acids were dissolved in 1000 µL petroleum ether (Sigma-Aldrich, Taufkirchen, Germany) followed by addition of 50 µL sodium methoxide-solution (2 mol/L (w:v) in methanol; Sigma-Aldrich, Taufkirchen, Germany) in accordance with Kohn et al. (33). After derivatization, the fatty acid methyl ester (FAME) fraction was completely evaporated under nitrogen and resolved in hexane (Sigma-Aldrich, Taufkirchen, Germany) to inject FAMEs into a gas chromatograph (Trace 1300, Thermo Scientific, Dreieich, Germany) equipped with an autosampler AS1310 (Thermo Fisher Scientific, Dreieich, Germany). The chromatographic separation of fatty acids was achieved using a capillary pre-column (GuardGOLD, length: 2 m, i.d.: 0.25 mm, Thermo Fisher Scientific, Dreieich, Germany) and downstream a capillary column named TRACE TR-FAME (70% cyanopropyl polysilphenylene siloxane, length: 60 m, i.d.: 0.25 mm, film thickness: 0.25 µm, Thermo Fisher Scientific, Dreieich, Germany). Samples were injected splitless at 250°C with 20 mL/min helium as the carrier gas (purification 99%, Air Liquide Deutschland GmbH, Düsseldorf, Germany). FAMEs were detected by a flame-ionization detector (FID) at 250 °C. Fatty acids were identified by their retention times (RT) compared with the RT of fatty acids in the external standard. The external standard was composed of the Supelco 37-Component FAME mix (Sigma-Aldrich, Taufkirchen, Germany) plus seven methylated single standards: vaccenic acid (VAC; C18:1n-7), stearidonic acid (SDA; C18:4n-3), nonadecanoic acid (C19:0; as internal standard), mead acid (MA; C20:3n-9), adrenic acid (ADA; C22:4n-6), docosapentaenoic acid (DPA; C22:5n-3) and palmitoleic acid (C16:1n-7t). VAC, C19:0, MA, ADA and n-3 DPA standards were all purchased from Sigma-Aldrich (Taufkirchen, Germany), whereas SDA and C16:1n-7t standards were purchased from Biomol (Hamburg, Germany). The column oven program was as follows: After injection, 60°C was maintained for 0.5 min whereafter temperature was increased to 180°C within 40 min. Then, the temperature was maintained constantly at 180°C for 2 min. Afterwards, the temperature was elevated to 210°C within 2 min to become constant at 210°C for 3

minutes. Finally, the temperature was increased to 240°C within 3 minutes to be constant at 240°C for 10 min. The total running time of the chromatographic process was 44 min.

Free fatty acid examination

Free fatty acids were determined in parallel with lipid mediators by liquid chromatography coupled with tandem mass-spectrometry (see Methods). For quantification, standard curves of fatty acid standards and deuterated (d) d_8-5S-hydroxyeicosatetraenoic acid (as internal standard) were used.

Macrophage polarization experiments

Monocytes were isolated from buffy coats purchased from the German Red Cross Blood Service (Frankfurt am Main, Germany, ethics committee vote 329/10) by ficoll separation and an indirect magnetic labelling system (Monocyte isolation kit II, Miltenyi Biotech, Germany). Cells were seeded at 5×10^5 cells/mL in 6-well plates and pre-incubated with PGE_2, PGE_3, LTB_4 and LTB_5 at 1000 nM for 30 minutes before differentiation as described before (1, 2). Monocytes were either differentiated in M1-macrophages (7 days 20 ng/mL GM-CSF following 24 h 20 ng/mL IFN-γ and 1 ng/mL LPS) or M2-macrophages (7 days 20 ng/mL M-CSF following 48 h 20 ng/mL IL-4) started at 37 °C and 5% CO_2. Medium with differentiation reagents and lipid mediators were renewed every other day. After the differentiation time supernatants were removed and cells were washed with PBS and detached with accutase (Sigma-Aldrich, Taufkirchen, Germany) for 15 min at 37 °C and 5%CO_2. Detached cells were washed with Stain Buffer (PBS + 2% FCS) and stained with CD14-APC, CD86-FITC, CD206-PE, CD64-FITC, HLA-DR-PerCpCy5.5, CD163-PE (BD Pharmingen), CD54-FITC (BioLegend, Dedham, USA), CD11c-PE and CD11b-FITC (Immunotech, Marseille, France). After washing with PBS, cells were resolved in 400 µL PBS and measurement was performed using the FACSVerse flow cytometer (BD Biosciences, Heidelberg, Germany) and the FACSuite software v1.0.6 (BD Biosciences, Heidelberg, Germany). Induction of M1- and M2- phenotypes after differentiation are illustrated in Sup. Figure 1.

Sup. Figure 1:

Cell surface marker expression of $CD14^+$ monocytic cells differentiated into either M1- or M2-macrophages. Histograms of CD14, CD86, CD11b, CD54, CD64, CD11c, CD206, CD163, HLA-DR expression on A) M1-macrophages and B) M2-macrophages (black line = stained control, red line = stained sample). Data represent one out of n = 6 experiments. C) The percent change of markers after differentiation into either M1- or M2-macrophages. Values are represented as mean ± SEM (n = 6). Differences were considered statistically significant at p-values < 0.05. * $p < 0.05$, ** $p < 0.01$, *** $p < 0.001$ tested by a RM-one-way ANOVA with Holm-Sidak's post-hoc analysis.

Sup. Figure 2:

Impact of 1000 nM PGE_2 or PGE_3 on macrophage polarization. Cell surface marker expression of CD86, CD11b, CD54, CD11c, CD206 and HLA-DR are represented as fold changes (FC) ± SEM (n = 3). Differences were considered statistically significant at p-values < 0.05. * $p < 0.05$, ** $p < 0.01$, *** $p < 0.001$ tested by a Friedman test with Dunn's post-hoc analysis.

Sup. Figure 3:

Impact of 1000 nM LTB_4 or LTB_5 on macrophage polarization. Cell surface marker expression of CD86, CD11b, CD54, CD11c, CD206 and HLA-DR are represented as fold changes (FC) ± SEM (n = 3). Differences were considered statistically significant at p-values < 0.05. * $p < 0.05$, ** $p < 0.01$, *** $p < 0.001$ tested by a Friedman test with Dunn's post-hoc analysis.

Sup. Table 1: Total and free fatty acid profiles

Footnotes Sup. Table 1: A) Total fatty acids of lung cells measured by capillary gas chromatography (C-GC). Results are presented as mean ± SEM; n = 5-10 mice per group and presented as percentage of total determined fatty acid quantities. B) Free fatty acids in lung cells measured by liquid chromatography coupled with tandem mass spectrometry (LC-MS-MS). Results are presented as mean pg/100 mg lung tissue ± SEM, n = 3-5 mice per group. Differences were tested by one-way ANOVA with Dunnett's post-hoc test considered statistically significant at p-values < 0.05. * $p < 0.05$ ** $p < 0.01$ and ***$p < 0.001$ and unpaired t-test (HDM + LCPUFA vs. HDM + EPA) considered statistically significant at p-values < 0.05. * $p < 0.05$ ** $p < 0.01$ and ***$p < 0.001$.

Sup. Table 2: Lipid mediator profiling

Footnotes Sup. Table 2: Quantities of all lipid mediators determined by lipid mediator profiling via liquid chromatography coupled with tandem mass spectrometry in lung tissue of non-supplemented asthmatic (HDM) or asthmatic mice supplemented with the LCPUFA combination (HDM + LCPUFA) or EPA (HDM + EPA) alone. Results are presented as mean pg/100 mg lung tissue ± SEM; $n = 3–5$ mice per group. Differences were tested by one-way ANOVA with Dunnett's post hoc test considered statistically significant at p-values < 0.05. * $p < 0.05$ ** $p < 0.01$ and ***$p < 0.001$ and unpaired t-test (HDM + LCPUFA vs. HDM + EPA) considered as statistically significant when p-values < 0.05. * $p < 0.05$ ** $p < 0.01$ and ***$p < 0.001$.

Literature:

1. Pistorius K, Souza PR, Matteis R de, Austin-Williams S, Primdahl KG, Vik A et al. PDn-3 DPA Pathway Regulates Human Monocyte Differentiation and Macrophage Function. Cell Chem Biol 2018; 25(6):749-760.e9.

2. Dalli J, Serhan CN. Specific lipid mediator signatures of human phagocytes: Microparticles stimulate macrophage efferocytosis and pro-resolving mediators. Blood 2012; 120(15):e60-72.

Sup. Table 1:

A) total FA [%]	CTRL (n=10)			HDM (n=10)			HDM + LCPUFA (n=10)			HDM + EPA (n=5)		
	Mean	±	SEM	Mean	±	SEM	Mean	±	SEM	Mean	±	SEM
**EPA**	0.06	±	0.01	0.08	±	0.01	1.44	±	0.15***/ ***	2.64	±	0.17***
**n-3 DPA**	0.87	±	0.05	0.87	±	0.03	4.82	±	0.26***/ p=0.07	6.10	±	0.57***
**DHA**	3.55	±	0,26**	4.51	±	0.13	5.91	±	0,15**/ ***	3.06	±	0,41**
**AA**	11,34	±	0.52***	13,86	±	0.36	8.96	±	0.38***/ ***	7.24	±	0.29***

B) free FA [pg/ 100mg]	CTRL (n=5)			HDM (n=5)			HDM + LCPUFA (n=5)			HDM + EPA (n=3)		
	Mean	±	SEM	Mean	±	SEM	Mean	±	SEM	Mean	±	SEM
**EPA**	847.00	±	233.90	1813.00	±	351.50	4970.00	±	453.40***	6162.00	±	228.80***
**n-3 DPA**	1565.00	±	298.30	2269.00	±	868.90	6470.00	±	854.80**	8480.00	±	808.20***
**DHA**	3184.00	±	503.20	2570.00	±	454.00	3300.00	±	269.20	4090.00	±	462.40
**AA**	7481.00	±	2079.00	6462.00	±	1320.00	6651.00	±	1472.00	6669.00	±	1311.00

Sup. Table 2:

	MRM tran-sitions		HDM (n=5)			HDM + LCPUFA (n=5)			HDM + EPA (n=3)		
	Q1	Q3	Mean	±	SEM	Mean	±	SEM	Mean	±	SEM
**RvD1**	375	215	42.49	±	6.50	46.65	±	5.99*	21.05	±	4.09
**RvD2**	375	141	2.12	±	1.07	4.53	±	2.01	4.83	±	1.53
**RvD3**	375	147	0.21	±	0.03	0.37	±	0.12*	0.10	±	0.05
**RvD4**	375	225	51.57	±	5.84	61.06	±	9.27*	23.68	±	3.49*
**RvD5**	359	199	13.32	±	4.39	22.85	±	7.72	24.23	±	5.49
**RvD6**	359	159	25.87	±	7.85	38.14	±	11.35	39.71	±	9.05
**17R-RvD1**	375	215		-		0.25	±	0.25	0.18	±	0.18
**17R-RvD3**	375	147	7.44	±	1.20	8.87	±	0.90*	4.07	±	0.74
**PD1**	359	153	2.53	±	0.31	4.26	±	1.15	4.77	±	1.48
**10S,17S-diHDHA**	359	153	193.90	±	56.72	278.00	±	85.82	290.40	±	65.63
**17R-PD1**	359	153	41.27	±	11.49	20.69	±	4.16	27.86	±	8.05
**22-OH-PD1**	375	137	0.86	±	0.20	0.58	±	0.17	0.25	±	0.04*
**PCTR1**	650	231	468.50	±	116.40	765.10	±	311.40	701.80	±	137.20
**PCTR2**	521	231	4.29	±	1.14	4.36	±	2.56	4.76	±	1.79
**PCTR3**	464	231	2.58	±	0.73	3.54	±	1.48	2.09	±	1.62
**MaR1**	359	177		-			-			-	
**MaR2**	359	177	12.66	±	2.12	25.22	±	6.33	26.28	±	5.86
**22-OH-MaR1**	375	221		-		0.33	±	0.21	0.55	±	0.28
**14-oxo-[illegible]**	357	221	0.02	±	0.02	0.14	±	0.07	0.11	±	0.00
**7S,14S-diHDHA**	359	248	5.64	±	1.40	12.14	±	3.46	10.45	±	2.49
**4S,14S-diHDHA**	359	159	44.85	±	13.89	65.38	±	21.13	68.24	±	15.78
**MCTR1**	650	191	370.80	±	112.70	770.40	±	351.00	700.10	±	227.40
**MCTR2**	521	191	3.24	±	0.62	5.85	±	2.78	6.41	±	2.12
**MCTR3**	464	191	4.84	±	2.91	5.13	±	1.37	4.21	±	1.89
**RvT1**	377	211	34.83	±	11.11	52.05	±	9.75	45.19	±	12.27
**RvT2**	377	197	0.03	±	0.03	0.11	±	0.07	0.02	±	0.02
**RvT3**	377	193	1.47	±	0.14	6.34	±	1.79**	4.50	±	1.35
**RvT4**	361	209	31.45	±	1.81	141.70	±	14.17*	141.10	±	16.12*
**RvD1$_{n-3\ DPA}$**	377	215	81.75	±	11.45	55.45	±	10.50	35.60	±	7.24*
**RvD2$_{n-3\ DPA}$**	377	233	0.08	±	0.08	0.20	±	0.20	0.11	±	0.11
**RvD5$_{n-3DPA}$**	361	199	27.25	±	4.17	174.20	±	39.81	147.90	±	21.56
**PD1$_{n-3DPA}$**	361	183	0.38	±	0.14	1.39	±	0.78	0.95	±	0.95
**10S,17S-diHDPA**	361	155	3.71	±	1.41	28.98	±	8.74	53.78	±	16.21*

**MaR1$_{n\text{-}3DPA}$**	361	223		-			-			-	
**7S,14S-diHDPA**	361	223	7.16	±	1.75	37.58	±	8.99*	34.55	±	4.18*
**RvE1**	349	195	1.93	±	0.97	2.63	±	1.10	5.25	±	2.31
**RvE2**	333	159	1.07	±	1.00	13.52	±	3.78	26.78	±	5.28**
**RvE3**	333	201	0.49	±	0.39	8.88	±	2.09*	9.49	±	5.15
**LTC$_5$**	624	187	2.75	±	0.93	33.32	±	12.32*	315.50	±	112.30**
**LTD$_5$**	450	187	40.11	±	40.11	35.02	±	17.42	47.71	±	47.71
**LTE$_5$**	438	187	2.48	±	1.03	31.48	±	15.66	220.70	±	75.58**
**LTB$_5$**	333	199	0.15	±	0.08	2.31	±	0.98	7.02	±	1.78**
**PGE$_3$**	349	189	3571.00	±	197.30	11515.00	±	2992.00	28199.00	±	8991.00**
**TxB$_3$**	367	169	4214.00	±	1181.00	22333.00	±	5335.00*	47627.00	±	23028.00**
**LXA$_4$**	351	115	0.18	±	0.08	0.46	±	0.32	0.62	±	0.23
**LXB$_4$**	351	115	0.98	±	0.45	2.74	±	0.40*	2.52	±	0.36
**5S,15S-diHETE**	335	235	240.50	±	41.70	191.60	±	45.47	178.10	±	51.76
**15-epi-LXA$_4$**	351	115	14.01	±	1.99	10.43	±	2.24	4.62	±	0.72*
**15-epi-LXB$_4$**	351	221	161.60	±	22.97	120.40	±	25.80	53.32	±	8.29*
**13,14-dihydro-15-oxo-LXA$_4$**	351	115	6.31	±	0.51	50.36	±	14.78*	40.97	±	11.22
**15-oxo-LXA$_4$**	349	115		-		0.50	±	0.15*	0.48	±	0.14*
**LTB$_4$**	335	195	31.76	±	11.82	34.08	±	3.75	43.36	±	13.80
**5S,12S-diHETE**	335	195	31.28	±	11.88	32.15	±	3.99	41.94	±	13.65
**Δ6-trans-,12-epi-LTB$_4$**	335	195	19.77	±	3.70	15.00	±	5.59	25.89	±	9.91
**Δ6-trans-LTB$_4$**	335	195	23.42	±	4.56	20.01	±	7.15	32.02	±	13.24
**20-OH-LTB$_4$**	351	195	0.16	±	0.07	0.19	±	0.07	0.16	±	0.14
**LTC$_4$**	626	189	744.00	±	569.30	223.80	±	100.00	283.90	±	109.40
**LTD$_4$**	497	189	84.77	±	67.22	19.01	±	7.17	32.88	±	9.37
**LTE$_4$**	440	189	133.80	±	88.50	55.32	±	9.82	78.73	±	14.35
**EoxinC$_4$**	626	205	85.21	±	43.28	4.21	±	1.89*	37.15	±	14.56
**EoxinD$_4$**	497	205	1.17	±	0.61	0.00	±	0.00	0.00	±	0.00
**EoxinE$_4$**	440	205	12.43	±	7.25	6.95	±	2.48	59.91	±	46.38
**PGD$_2$**	351	189	6149.00	±	743.10	4377.00	±	442.40	3690.00	±	671.90*
**PGE$_2$**	351	189	8343.00	±	811.30	5372.00	±	889.90	4361.00	±	424.80
**PGF$_{2\alpha}$**	351	193	5723.00	±	149.50	2755.00	±	55.27*	2145.00	±	464.80*
**TxB$_2$**	369	169	5933.00	±	700.60	3311.00	±	596.60*	2981.00	±	525.50
